# Genetic Variation in Response to the Mediterranean–DASH Intervention for Neurodegenerative Delay (MIND): A Randomized Controlled Trial

**DOI:** 10.3390/nu18030508

**Published:** 2026-02-02

**Authors:** Marilyn C. Cornelis, Lisa L. Barnes

**Affiliations:** 1 Department of Preventive Medicine, Northwestern University Feinberg School of Medicine, Chicago, IL 60611, USA; 2 Department of Neurological Sciences, Rush University Medical Center, Chicago, IL 60612, USA; 3 Rush Alzheimer’s Disease Center, Chicago, IL 60612, USA

**Keywords:** diet pattern, dementia, genotype, cognitive decline, clinical trial

## Abstract

**Background**: The Mediterranean–DASH Intervention for Neurodegenerative Delay (MIND) study was a 3-year randomized controlled trial to test the effects of the MIND diet on cognitive decline in individuals at risk for Alzheimer’s dementia (AD). Here we examine whether genetic differences in (a) AD predisposition and (b) nutrient metabolism modify the effect of MIND on cognitive change. **Methods**: This secondary analysis included 494 trial participants of genetically inferred European ancestry with genetic data. Genetic scores (GS) were derived from prior genome-wide studies of AD and nutrient biomarkers. Linear regression and linear mixed models were used to examine the main effects of GS and interactions with diet assignment on cognition. An exploratory genome-wide interaction analysis was also performed. **Results**: We observed a statistically significant interaction (*p* = 0.002) between the *COMT* Val158Met variant and diet assignment for the 3-year change in perceptual speed. *Met*/*Met* (lower enzyme activity) carriers’ perceptual speed improved more on the MIND than the control diet, while no difference by diet was observed for *Val* carriers. **Conclusions**: Catechol-O-methyltransferase (COMT) metabolizes catecholamines as well as polyphenols unique to the MIND diet. Individuals with genetically impaired COMT activity may be especially responsive to the cognitive benefits of the MIND diet.

## 1. Introduction

The Mediterranean–DASH Intervention for Neurodegenerative Delay (MIND) diet is based on the most compelling evidence in the diet–dementia field [1,2]. The MIND diet emphasizes consuming green leafy vegetables, olive oil, berries, nuts, legumes, whole grains, seafood, poultry, wine, and limited intake of animal and high-saturated-fat foods (Table S1). Population studies suggest adherence to the diet may slow the rate of cognitive decline and decrease the risk of dementia [3]. The first and longest randomized controlled trial (RCT) of a calorie-restricted MIND diet on cognitive change reported no significant differences in cognitive change after 3 years compared to a calorie-restricted control diet. Both arms lost weight and presented with significant improvements in cognition, thus potentially emphasizing a key role of weight management in brain health [4]. The latter is also supported by results of two smaller and shorter RCTs of a calorie-restricted MIND diet [5,6].

The rate of age-associated cognitive decline varies among individuals, and this variation likely arises from a complex interplay of genetic and environmental factors. Prior studies have examined *APOE* ε4 and comprehensive AD genetic scores as potential modifiers of the MIND diet with cognitive outcomes, but findings have been mixed [4,7,8,9,10,11]. Genetic variation in the metabolism of constituents characteristic of the MIND diet may enhance or limit their efficacy, but has not been examined. For example, genetic variants have been identified for nutrient biomarkers that correlate with MIND foods such as vitamin K (greens), vitamin E (olive oil, nuts, greens), carotenoids (other vegetables), polyphenols (berries, olive oil), folate (beans, greens, other vegetables), and oleic and azelaic acid (olive oil) (Table S2), which might in turn modify anti-inflammatory, antioxidant, vascular or neural responses to MIND that benefit cognitive function [12]. The current study is a secondary analysis of the 3-year MIND RCT, which examines whether genetic differences in AD predisposition and nutrient metabolism modify the effect of the MIND diet on cognitive change. Considering the results of MIND RCTs and the role of insulin resistance and obesity in cognitive decline [13,14,15,16], genetic susceptibility to these disorders was also considered. A genome-wide analysis of the effect of MIND on cognitive change was explored to provide a data resource for other nutrition or genetic investigators.

## 2. Materials and Methods

### 2.1. The MIND Trial

The MIND trial design and results from the primary analysis have been published previously [4,17]. This 3-year RCT examined the effect of a calorie-restricted MIND diet compared to a calorie-restricted usual diet (control diet) on cognitive decline. The trial had two clinical sites: Chicago (Rush University) and Boston (Harvard University). Men and women aged 65+ years who were cognitively unimpaired, overweight, and had a family history of dementia and a suboptimal diet (MIND score ≤ 8 out of 14) were recruited. Those determined eligible completed a 3- to 4-week run-in to assess their likelihood of complying with trial interventions. Participants who successfully completed the run-in were invited back to complete a 2-day baseline visit. Measures of height, weight, and a fasting blood sample were collected, cognition tests were performed, and questionnaires were completed. All participants were randomly assigned in a 1:1 ratio to the MIND-diet group or the control-diet group, stratified by trial site, sex, and age. Both assigned diets had mild calorie restriction (250 kcal deficit/d) to achieve a weight loss of 3–5% by year 3. The MIND diet is detailed in Table S1. While the original MIND diet encourages up to 1 glass of wine per day, no wine recommendations were made for the trial. The control diet consisted of the usual diet. MIND-diet participants were supplied with mixed nuts (5 oz/week), extra virgin olive oil (14 tbsp/week), and blueberries (2.5 cups/week). Control-diet participants received USD 30 gift cards per week. All clinical measures, data collection, and biospecimen handling were performed by trained clinical staff. During the COVID-19 pandemic, in-person visits were halted for ~3 months, but dietary counseling by telephone continued. After stay-at-home orders were lifted, 24 participants in the MIND-diet group and 20 in the control-diet group declined to return for an in-person visit.

The trial followed the ethical standards of the 1964 Declaration of Helsinki and its later amendments and was approved by the institutional review boards at Rush University Medical Center, Harvard School of Public Health, and Brigham and Women’s Hospital. Participants were enrolled from January 2017 to April 2018; data collection ended in June 2021. A total of 604 participants were enrolled and randomized. All the participants provided written informed consent.

### 2.2. Cognitive Battery

The primary endpoint of the MIND trial was the change from baseline to year 3 in a global cognition score and four cognitive domain scores. A battery of 12 cognitive tests was administered during clinical visits at baseline, 6 months, 12 months, 24 months, and 36 months. Cognitive testing was conducted by research assistants who were certified in cognitive testing and blinded to diet assignments. Tests for episodic memory, semantic memory, perceptual speed, and executive function are listed in Table S3. The raw scores from each test were converted to z-scores, which were averaged across all tests to create the global cognition score and across component tests to create the four domain scores; higher scores indicate better cognitive performance. Participants who did not return for in-person visits in year 3 completed episodic and semantic memory tests by telephone.

### 2.3. Genetic Data and Calculation of Genetic Scores (GS)

Genotyping, imputation, and quality control (QC) procedures for the genetic data of trial participants have been described in detail previously [18]. Briefly, DNA was extracted from either whole blood or serum, and genotyping was performed using the Infinium Global Diversity Array. Established sample and SNP QC procedures were applied to the genotyping data, followed by imputation using the 1000 Genomes Phase 3 v5 reference panel. A total of 494 unrelated individuals of inferred European ancestry and 58 unrelated individuals of inferred African ancestry were included in the final imputed dataset. The current analysis is limited to the 494 participants of European ancestry to avoid population stratification, and since sample sizes of other ancestries were too small for meaningful analysis. All SNPs initially selected for genetic scores (GS) were genome-wide significant (*p* < 5 × 10^−8^, GS_vitK_ the exception), biallelic and common (MAF > 1%) in GWAS of European ancestry. We then excluded SNPs poorly imputed (R^2^ < 0.8) in the MIND trial sample and that lacked high-quality proxies (1 SNP for folate and 1 SNP for IR). GS for AD (GS_AD_) was computed as described previously using 25 SNPs (excluding *APOE* SNPs, Table S4) [9,19,20]. GS_AD_ uses the sum of the products of SNP risk alleles and their corresponding weights as GS_AD_ = Σ*_i_*^n^ log(OR*_ij_*) × G*_ij_* for the *i*th individual, where log(OR*_ij_*) = the log of the OR for the *j*th SNP, G*_ij_* = the number of risk alleles (0, 1, or 2) for the *j*th SNP, and *n* = 25 candidate SNPs. The score is rescaled according to the number of risk alleles (#SNPs×2) to facilitate interpretation. A higher GS_AD_ corresponds to an elevated AD risk. GS_AD-I_ and GS_AD-C_ are subset scores of GS_AD_ and include SNPs mapping to, respectively, the immune response (10 SNPs) and cholesterol metabolism (4 SNPs) pathways (Table S4). *APOE* was not included in the GSs because of its large effect size, and it was previously examined separately in the main MIND trial results [4]. The same scoring method (using beta coefficients in place of logOR if possible) was used to generate GS for vitamin K (phylloquinone, 4 SNPs, GS_vitK_), vitamin E (alpha + gamma tocopherols, 5 SNPs, GS_vitE_), beta-carotene (1 SNP, GS_βcar_), alpha-carotene (3 SNPs, GS_αcar_), folate (1 SNP, GS_folate_), brain Fe (12 SNPs, GS_Fe_), azelaic acid (1 SNP, GS_azelaic_) and oleic acid (1 SNP, GS_oleic_) using results of corresponding biomarker GWAS (Table S2). Finally, a GS for BMI (75 SNPs, GS_BMI_) [21] and insulin resistance (28 SNPs, GS_IR_) [22] was also derived.

### 2.4. Statistical Analysis

All statistical analyses were performed using the SAS v9.4 statistical package (SAS Institute, Cary, NC, USA) and PLINK 2.0.

Hypothesis testing: We first examined the main effects of GS (modeled additively/continuously) on baseline cognition and cognitive change independent of diet assignment using linear regression and linear mixed models, respectively. Linear regression models included terms for GS, age, sex, study site, specimen, and the top 10 PCs. Linear mixed models included terms for GS, age, sex, study site, DNA specimen, and the top 10 PCs as fixed effects; intercept and time (visit, slope) as random effects; and multiplicative terms between time and each model covariate. Mixed models included all available data, including those from participants with incomplete follow-up; missing data were not imputed and assumed to be missing at random. As per the original trial analyses, trajectories of cognition across time were nonlinear and thus time was modeled as an indicator variable for each visit. Within-person residuals were assumed to follow a first-order autoregressive model that was conditional on the random intercepts. The GS × time term estimated mean differences in rates of cognitive change with increasing GS. Participants with no follow-up visits (N = 13) contributed to the estimation of fixed effects and the overall mean but did not contribute to estimating within-person variance or random effects. Mean cognition at baseline and mean change in cognition from baseline by GS were computed using estimated marginal means. We then proceeded to test for GS × diet interactions on cognitive change by adding diet assignment and cross-product terms of diet × time, and GS × diet × time to mixed models; the latter providing an estimate of the interaction for cognitive change. Formal interaction testing was complemented by tabulating mean cognitive change from baseline stratified by diet assignment and GS tertiles (or genotype for GS composed of a single SNP). We tested 14 GS for interactions with diet on change in global cognition, and specific domains of episodic memory, executive function, perceptual speed, and semantic memory. A Bonferroni correction was used to account for 14 GS tested: *p*-values less than 0.004 (=0.05/14 tests) were considered statistically significant. We did not correct for the number of cognition measures since they were generally correlated. Nevertheless, we applied a post hoc false discovery rate (FDR) correction to evaluate the robustness of our interaction results. For significant interactions, we further examined whether results persisted when adjusting for weight change or if the interacting GS was associated with diet adherence or loss to follow-up. We also explored associations between GS and spot urine measures of tyrosol (corrected for creatinine) available in a subset of our analytical sample at baseline, 3 months, 12 months, 24 months, and 36 months [23]. We analyzed participants with baseline and at least one follow-up measure of tyrosol and excluded only two outlying data points (one from each diet arm at 12 months) that were over 19-fold higher than the sample mean. Linear mixed models, as described above for cognitive change, were also applied for urinary tyrosol change with additional adjustment for batch.

Hypothesis generating (exploratory): Two approaches to the genome-wide (GW) diet interaction analysis were performed. First, mixed effects models described above, but excluding GS and diet assignment, were used to estimate individual paths of change in cognition. Unlike our hypothesis testing approach described above, time was modeled linearly to avoid downstream modeling of multiple paths (per visit) per individual. Residuals, individual slopes, were extracted from these models and used as quantitative outcome phenotypes for GW interaction testing using linear regression models. Predictor variables included SNP, diet assignment, and SNP × diet assignment. The second approach used each participant’s change in cognition from baseline to 36 months as the outcome for GW interaction testing and further adjusting for covariates listed for mixed models described above. For individuals with missing test scores at 36 months (N = 31 for global, semantic, and episodic, 63 for perceptual, and 64 for executive), we carried forward their last test score. We considered results for SNPs with MAF > 1% and high imputation quality scores (R^2^ > 0.8), leaving 7,647,009 SNPs for analysis. We applied a genome-wide interaction significance threshold of *p* < 5 × 10^−8^ for either approach, but considered only results that were consistent across approaches as promising.

## 3. Results

Baseline characteristics of the MIND trial participants used in the current analysis are presented in Table 1. As expected, the self-identified race of our sample (selected on European ancestry) differed from the full trial sample.

### 3.1. Hypothesis Testing

None of the GS were significantly associated with baseline measures of cognition (*p* > 0.004). Nominal associations were observed between GS_azelaic_ and executive (*p* = 0.04), GS_BMI_ and global (*p* = 0.03), GS_COMT_ and executive (*p* = 0.03), and GS_folate_ and semantic (*p* = 0.02) (Table S5). Greater genetic susceptibility to higher blood azelaic acid, BMI, and blood folate was associated with better baseline performance on executive function, global cognition, and semantic memory tests, respectively. Higher genetically inferred COMT expression (GS_COMT_, *Val*/*Val*) was associated with lower executive function.

**Figure 1:**
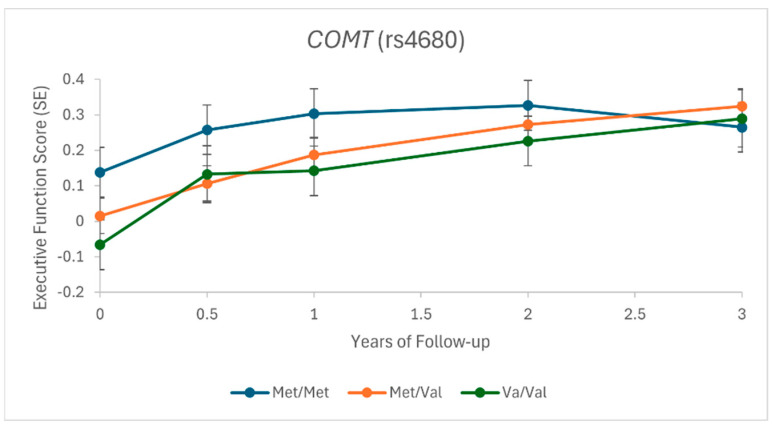
GS_COMT_ (rs4680) and change in executive function. Shown are the estimated means of executive function score by genotype from linear mixed models (*p* = 0.02).

In linear mixed models, none of the GS were significantly associated with changes in cognition after 3 years (*p* > 0.004). Higher genetically inferred blood azelaic acid (GS_azelaic_) nominally associated with less improvement in executive function (*p* = 0.009) (Figure S1, Table S6), while higher genetically inferred COMT expression (GS_COMT_, *Val*/*Val*) was nominally associated with greater improvement in executive function (*p* = 0.02) (Figure 1, Table S6).

**Figure 2:**
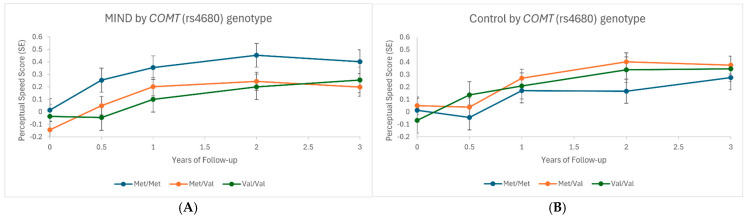
(**A**,**B**) GS_COMT_ (rs4680) and change in perceptual speed by diet assignment. Shown are estimated means of perceptual speed score by genotype and diet assignment from linear mixed models (*p* = 0.002 for interaction).

We observed a statistically significant GS_COMT_ × diet × time interaction (*p* = 0.002) for the 3-year change in perceptual speed, whereby *Met*/*Met* (lower COMT expression) carriers performed better on MIND (Figure 2A) than the control diet (Figure 2B and Table S7). From baseline through year 3, the estimated mean (95% CI) change in perceptual speed score was 0.18 (0.07, 0.30) standardized units in Met/Met carriers on the MIND diet and 0.03 (−0.13, 0.18) standardized units in Met/Met carriers on the control diet. Conversely, no difference in change in perceptual speed over time by diet was observed for *Val* carriers. This interaction was not significant (q value = 0.11) when applying an FDR threshold of 0.05. In post hoc analysis, the interaction persisted (*p* = 0.002) when models were further adjusted for weight change. GS_COMT_ was not associated with a change in MIND diet adherence overall or among the MIND diet arm specifically (*p* > 0.05). GS_COMT_ was also not associated with the number of follow-up visits (*p* > 0.05). Measures of urinary tyrosol (corrected for creatinine), a polyphenol and biomarker of olive oil intake, were available for 388 participants (172 MIND arm, 166 control arm) of our analytical sample. GS_COMT_ was not associated with baseline (i.e., pre-intervention) tyrosol (*p* > 0.05) or change in tyrosol after 3 years (*p* > 0.05). A nominal GS_COMT_ × diet × time interaction (*p* = 0.03) for the 3-year change in tyrosol was observed, but was not interpretable upon visualization (Figure S2). No evidence of interaction was observed when truncating outlying tyrosol measures to 4 SD of the mean (*p* > 0.05).

### 3.2. Hypothesis Generating

The union of two approaches to GW interaction testing yielded genome-wide signals for change in global cognition (1 locus), executive function (17 loci), perceptual speed (1 locus), and semantic memory (9 loci) (Table S8, https://prism.northwestern.edu/, Figures S3–S6). Index SNP allele frequencies ranged from 0.01 to 0.09; 16 were intronic, 11 were intergenic, while the remaining were in untranslated regions. Mean change in cognitive scores from baseline stratified by genotype and diet assignment confirmed that most interactions were driven by extreme scores among the very few heterozygotes and homozygote minor allele carriers (Table S9).

## 4. Discussion

In this secondary analysis of the 3-year MIND RCT, we examined whether genetic differences in AD predisposition and nutrient metabolism modify the effect of the MIND diet on cognitive change. We confirm our prior population studies showing that genetic differences in AD susceptibility do not modify the association between MIND adherence and cognitive decline [8,9]. To our knowledge, this is the first study to examine whether genetic differences in MIND-specific nutrient metabolism impact the effect of the MIND diet on cognition over time. Our primary findings pertain to a well-characterized *COMT* variant, which presented both main effects and interactions with diet assignment on cognition.

The MIND diet is rich in polyphenols due largely to the olive oil, berries, and other fruits and vegetables specified [24,25,26]. In the MIND diet trial, extra virgin olive oil and blueberries were provided to participants assigned to the MIND diet to promote adherence to the diet. Olive oil and blueberries are rich sources of hydroxytyrosol and anthocyanins, respectively, known for their antioxidant, anti-inflammatory, and neuroprotective properties [27,28]. *COMT* encodes catechol-O-methyltransferase, an enzyme that metabolizes catecholamines as well as polyphenols such as hydroxytyrosol, anthocyanins, and flavan-3-ols (i.e., catechins) [29]. Our GS_COMT_ includes one SNP (rs4680) resulting in a Val158Met substitution in *COMT* [30]. A progressive reduction in COMT protein and COMT enzymatic activity occurs in a *Met* dose–response manner, which results in higher synaptic dopamine [30,31,32,33,34]. Nominal associations were observed for GS_COMT_ with both baseline and 3-year change in executive function (independent of diet assignment). Our findings linking *COMT* rs4680 to executive function are consistent with prior studies [35,36] and key roles of the prefrontal cortex and dopamine in executive function [33,37]. *Val*/*Val* carriers had the lowest baseline score, but over 3 years, presented with the greatest score improvement. The lower baseline scores for *Val*/*Val* carriers might be more responsive to re-testing or weight loss effects (both diets), while test ceiling effects mask any additional improvement among *Met* carriers. In a small RCT, *Val*/*Val* carriers had lower memory scores than *Met* carriers at baseline but presented with greater improvements after 3 months of caloric restriction [38]. Methylation typically reduces the biological activity of a compound [29,39,40] and thus we hypothesized that *Met*/*Met* carriers (lower GS_COMT_) may perform cognitively better on the MIND diet than the control diet compared to *Val*/*Val* carriers (higher GS_COMT_). Indeed, we observed a statistically significant and novel GS_COMT_ × diet interaction for 3-year change in perceptual speed, whereby *Met*/*Met* performed better on the MIND diet than the control diet. No difference in change by diet was observed for *Val* carriers. The interpretation of the results is complicated by studies suggesting the main biological metabolite of hydroxytyrosol is the product of COMT: 3-O-methyl-hydroxytyrosol (also known as homovanillyl alcohol) [41,42,43]. In this scenario, higher COMT (*Val*/*Val*) activity would be preferred. Biomarkers serving as direct substrates or metabolites of COMT were not measured in the trial. Tyrosol is structurally related to hydroxytyrosol and is also present in olive oil, but is not a substrate/metabolite of COMT since it lacks the catechol structure [44]. However, it can be partially converted to hydroxytyrosol and thus could be a precursor [45]. Nevertheless, our analysis of urinary tyrosol in a subset of trial participants did not support our significant findings for COMT. Since COMT has multiple substrates and functions, deciphering the constituents of MIND driving the observed interaction warrants further study. Why this interaction was specific to perceptual speed (and not executive function) is also unclear. In a recent proteomic analysis of eight healthy diet patterns in the UK Biobank, COMT was significantly and inversely associated with all dietary patterns, but most strongly with MIND [46]. This suggests that MIND adherence may also modify the activity of COMT, but whether this association is causal is unknown.

Azelaic acid is a potential biomarker of olive oil, berry, and whole grain intake [47,48,49]. It is also a metabolic byproduct of *Malassezia furfur*, a yeast found on human skin, and is used topically in many skincare products [50]. Our GS_azelaic_ contains one SNP in the intronic region of *SLC17A1*; the A allele associates with higher blood azelaic acid levels compared to the G allele in GWAS [51]. While only nominally significant, baseline executive function increased with each A allele, and after 3 years, participants homozygous for G (lowest performance at baseline) saw greater improvement in executive function; no interaction with diet assignment was observed. This pattern of results mirrors that of GS_COMT,_ and thus a similar interpretation holds. SLC17A1 is highly expressed in the kidney and functions in the transport of organic anions, in particular, urate [52]. Indeed, the A allele also associates with higher uric acid in GWAS [53] and thus may also be contributing to the nominal main effects we observed. The relationship between blood azelaic acid and cognitive function is inconsistent [54,55] while urinary azelaic acid is generally elevated in participants with AD, major depressive or bipolar disorders, compared to controls [56,57]. Conversely, uric acid levels are inversely associated with AD and Parkinson’s disease with dementia [58] and would be more consistent with our genetic findings. The main effects of *COMT* and *SLC17A1* variation on cognition over time may be plausible, but we acknowledge that neither variant associates with cognitive decline or dementia in GWAS. Moreover, we cannot discount a possible interaction with weight loss, observed in both diet assignments over time.

Genetic variation in vitamin K, vitamin E, beta carotene, alpha-carotene, folate, oleic acid, and iron metabolic pathways did not modify cognitive response to MIND over time. Diet interactions with genetic variation in BMI and IR were also non-significant, though participant selection criteria (overweight) may have biased results. Our hypothesis testing was limited by knowledge of biomarkers for MIND-diet intake and corresponding genetic loci identified in European populations. GS had a variable number of SNPs with variable effect sizes, likely contributing to differential power to detect interactions. A genome-wide interaction analysis of the MIND diet on cognitive change was also performed as an exploratory analysis and to provide a data resource for other nutrition or genetic investigators. While we identified genome-wide significant SNP-diet interactions, these were driven by low-frequency variants and may be false positives. We have made all summary statistics for this exploratory analysis publicly accessible for replication or follow-up analysis.

The use of a large, well-designed RCT with excellent adherence for a comprehensive genetic analysis of cognitive response to the MIND diet is a major strength of the current study. However, unlike population-based genetic analysis of MIND adherence and cognitive function, the sample size provides limited power, especially for discovery. Our study was also limited to highly adherent participants of European Ancestry with an elevated risk of dementia.

## 5. Conclusions

In summary, we observed an interaction between the *COMT* Val158Met variant and diet assignment for the 3-year change in perceptual speed. Individuals with genetically impaired COMT activity may be especially responsive to the cognitive benefits of the MIND diet. Reduced perceptual speed in older adults is an early and sensitive indicator of broader cognitive decline and loss of independence in daily living activities [59,60]. While the original RCT did not report significant overall differences in cognitive change after 3 years of consuming a MIND diet compared to the control diet, our genetic analysis suggests potential response heterogeneity that warrants confirmation.

## Figures and Tables

**Table 1:** Baseline characteristics of MIND trial participants *.

Characteristic	Current Sample		Full Trial Sample	
	MIND	Control	MIND	Control
	N = 249	N = 245	N = 301	N = 303
Age at randomization, years	69.8 (4.2)	70.1 (4.3)	69.8 (4.2)	69.9 (4.2)
Male, %	37	36	35	35
At least a college degree, %	78	81	76	80
BMI	33.6 (5.3)	33.6 (5.9)	33.8 (5.4)	34.0 (6.5)
Race, %				
White	100	100	87	88
Black	0	0	12	10
Other	0	0	1	2
Current smoker, %	3	2	3	3
Diabetes, %	14	13	15	15
MIND score	7.6 (1.9)	7.8 (1.8)	7.7 (1.9)	7.8 (1.8)
Study Site, %				
Rush	47	49	50	50
Harvard	53	51	50	50
APOE ε4-carrier, %	24	32	25	32

* MIND trial participants for the current analysis vs. all MIND trial participants. Data are means (SD) unless otherwise specified.

## Data Availability

Genetic data will be made available via NIAGADS, https://dss.niagads.org/, in accordance with NIH/NIA guidelines for genetic resource sharing. MIND trial phenotyping can be accessed upon approval at https://www.radc.rush.edu. Summary statistics are available at https://prism.northwestern.edu/.

## Supplementary Materials

The following supporting information can be downloaded at: https://www.mdpi.com/article/10.3390/nu18030508/s1. Table S1. MIND diet. Table S2. Candidate nutrient and biomarker loci. Table S3. Cognitive domains and tests used in the MIND Trial. Table S4. Late-Onset Alzheimer’s Disease Risk Loci. Table S5. Nominal associations (0.004 < *p* < 0.05) between genetic scores (GS) and baseline cognitive performance. Table S6. Estimated mean change (95% CI) in executive function from baseline by GS_azelaic_ and GS_COMT_. Table S7. Estimated mean change (95% CI) in perceptual speed from baseline by diet assignment and GS_COMT_. Table S8. Genome-wide SNP×diet interactions and change in cognitive function after 3 years. Table S9. Estimated mean change (SD) in cognitive scores at 3 years from baseline stratified by genotype and diet assignment. Figure S1. GS_azelaic_ (*SLC17A1*, rs1165213) and change in executive function. Figure S2. GS_COMT_ (rs4680) and change in urinary tyrosol by diet assignment. Figure S3. Manhattan plots for genome-wide association analysis of change in global cognition score. Figure S4. Manhattan plots for genome-wide association analysis of change in executive function score. Figure S5. Manhattan plots for genome-wide association analysis of change in semantic memory score. Figure S6. Manhattan plots for genome-wide association analysis of change in perceptual speed score. References [61,62,63,64,65,66,67,68] are cited in the Supplementary Materials
