# Supplementary material for: Genetic Variation in Response to the Mediterranean–DASH Intervention for Neurodegenerative Delay (MIND): A Randomized Controlled Trial
Source: Nutrients. 2026 Feb 2;18(3):508. doi: 10.3390/nu18030508 (PMC12899571; doi:10.3390/nu18030508)
Supplement: Supplementary file 1 [file nutrients-18-00508-s001.zip › nutrients-4081324-supplementary.pdf]

Supplementary Materials

Genetic variation in response to the Mediterranean-DASH Intervention for Neurodegenerative Delay (MIND): A randomized controlled trial

Marilyn C. Cornelis, Lisa L. Barnes

Table S1. MIND diet

**Foods to Eat or Avoid**	**Serving Goals***
Green leafy vegetables	At least 6 per week (1/2 – 1 cup)
Other vegetables	At least 1 per day (1/2 cup)
Nuts (mixed nuts and/or peanut butter)	5 oz per week
Berries	At least 2 per week (1/2 cup)
Beans/legumes	At least 3 per week (1/2 cup)
Whole grains	3 servings per day
Fish (not fried)	At least 1 per week (3-5 oz)
Poultry (not fried, white meat/skinless)	At least 2 per week (3-5 oz each)
Extra virgin olive oil	2 TB per day
Red and processed meat	Less than 4 per week (3-5 oz each)
Butter and stick margarine	1 pat (tsp) or less per day
Cheese (whole fat)	2 oz or less per week
Pastries, candy bars, sweets	Less than 5 per week
Fried foods and fast food	1 meal or less per week

***Green leafy vegetables:** Spinach, kale, collards, swiss chard, mustard greens, turnip greens, dandelion greens, arugula, endive, grape leaves, romaine lettuce
• 1 serving = 1 cup raw or ½ cup cooked
Other vegetables: Artichokes, asparagus, avocado, broccoli, brussel sprouts, cabbage, carrots, cauliflower, corn, eggplant, green beans, mushrooms, leeks, onions, okra, peas, pumpkin, snow peas, squash (including zucchini), sweet bell peppers, sweet potatoes (not white potatoes), tomatoes/tomato sauce
• 1 serving = ½ cup cooked/cut up
Nuts: 5oz per week of mixed nuts and/or peanut butter Berries: Blueberries, strawberries, raspberries, and other berries
• 1 serving = ½ cup
Beans/legumes: Black, pinto, cannellini, garbanzo, kidney, lima, red/white, navy, lentils, tofu, edamame, hummus
• 1 serving =½ cup
Whole grains: 1 slice dark bread, ½ dark bagel, ½ c brown rice/pastas, wild rice, quinoa, barley, buckwheat, bulgar, farro, kamut, millet, oats, rye, spelt, 3/4 cup whole grain cereal
Fish (not fried): Fresh or canned fish or seafood as main dish; dark meat or light meat fish, salmon, tuna or tuna sandwiches, mackerel, trout, sardines, anchovies, shellfish
• 1 serving = 3-5 ounces cooked
Poultry (not fried, white meat, skinless): Chicken or turkey as main dish baked, broiled, or grilled
• 1 serving = 3-5 ounces cooked without skin/bone
Red and processed meats: Cheeseburger, hamburger, beef tacos/burritos, hot dogs/sausages, bacon, roast beef or ham sandwich, salami, bologna, or other deli meat sandwich, beef (steak, roast) or lamb as main dish, pork or ham as main dish, meatballs or meatloaf
• 1 serving = 3-5 ounces cooked
Pastries & Sweets: Biscuit/roll, poptarts, cake, snack cakes/Twinkies, Danish/sweet rolls/pastry, donuts, cookies, brownies, pie, candy bars, other candy, ice cream, pudding, milkshakes/frappes
Fried foods and fast food: Fish and chips, fried sandwiches, fried chicken, French fries, onion rings, chicken nuggets, tacos, pizza, hot dogs, falafel, hush puppies

Table S2. Candidate nutrient and biomarker loci

Trait (MIND component target)	Ref	Closest *Gene*	Chr:Pos GRCh37	SNP_EA	OA	Effect β	SNP Type*	R2†
alpha carotene (fruits & vegetables)	[1]	*CAPN8*	1:223872051	rs12137025_C	T	0.19	I	0.98
alpha carotene (fruits & vegetables)	[1]	*LINC02500*	4:182178306	rs17830069_G	A	0.38	I	0.98
alpha carotene (fruits & vegetables)	[1]	*PRKCE*	2:46409466	rs2594495_A	G	0.37	I	0.89
beta carotene (fruits & vegetables)	[2]	*BC01*	16:81258987	rs12926540_C	T	[+]	I	0.91
Vitamin E (alpha tocopherol, olive oil, greens)	[2]	*ZPR1*	11:116648917	rs964184_G	C	[+]	T/I	1.00
Vitamin E (alpha tocopherol, olive oil, greens)	[2]	*TM6SF2*	19:19379549	rs58542926_C	T	[+]	T/I	1.00
Vitamin E (olive oil)	[3]	*CYP4F2*	19:15990431	rs2108622_T	C	[+]	T/I	1.00
Vitamin E (gamma tocopherol, olive oil, greens)	[4]	*MTFP1*	22:30831852	rs11705639_G	A	[+]	I	0.944
Vitamin E (gamma tocopherol, olive oil, greens)	[4]	*NKAIN3*	8:63883970	rs62508088_C	T	[+]	I	0.988
Folate (beans & legumes, whole grains, greens, vegetables)	[5]	*MTHFR*	1:11856378	rs1801133_G	A	[+]	T/I	0.999
Folate (beans & legumes, whole grains, greens, vegetables)	[5]	*FOLR3*	11:71849741	rs652197_C	T	[+]	I	**0.599**
Phylloquinone (vitamin K1, green leafy) ‡	[6]	*ZPR1*	11:116648917	rs964184_C	G	0.23	T/I	1.00
Phylloquinone (vitamin K1, green leafy) ‡	[6]	*CTNAA2*	2:79624988	rs4852146_C	T	0.19	T/I	1.00
Phylloquinone (vitamin K1, green leafy) ‡	[6]	*KCNK9*	8:140496242	rs4645543_C	T	0.42	I	0.95
Phylloquinone (vitamin K1, green leafy) ‡	[6]	*CYP4F2*	19:15990431	rs2108622_T	C	0.16	T/I	1.00
Blood COMT protein level<br>Anthocyanin (berries) and hydroxytyrosol (olive oil) methylation	[7]	*COMT*	22:19951271	rs4680_G	A	[+]	T/I	1.00
Blood azelaic acid (olive oil, berries, whole grains)	[8-11]	*SLC17A1*	6:25799676	rs1165213_A	G	[+]	I	0.99
Oleic acid / MUFA (olive oil)	[12]	*FADS1/TMEM258*	11:61557803	rs102275_C	T	[+]	T/I	1.00
Brain iron § (neurodegeneration)	[13]	*THRAP3*	1:36690748	rs41267275_T	C	[+]	I	0.87
Brain iron § (neurodegeneration)	[13]	*SORCS2*	4:7475163	rs76278520_C	G	[+]	I	0.99
Brain iron § (neurodegeneration)	[13]	*NAMPT*	7:105842968	rs35447353_G	A	[+]	T	1.00
Brain iron § (neurodegeneration)	[13]	*AXIN2*	17:63392730	rs72862451_C	A	[+]	I	0.47
Brain iron § (neurodegeneration)	[13]	*COQ5*	12:121223159	rs77431485_A	G	[+]	I	0.87
Brain iron § (neurodegeneration)	[13]	*STAM*	10:17756975	rs45470994_A	T	[+]	I	0.51
Brain iron § (neurodegeneration)	[13]	*SLC40A1*	2:190440809	rs34206448_T	C	[+]	T	1.00
Brain iron § (neurodegeneration)	[13]	*TFRC*	3:195776976	rs406271_C	T	[+]	T	1.00
Brain iron § (neurodegeneration)	[13]	*SLC39A8*	4:103188709	rs13107325_T	C	[+]	T	0.99
Brain iron § (neurodegeneration)	[13]	*HFE*	6:26093141	rs1800562_A	G	[+]	T	1.00
Brain iron § (neurodegeneration)	[13]	*HLA-B*	6:31333032	rs9266366_T	C	[+]	I	0.96
Brain iron § (neurodegeneration)	[13]	*MAFK*	7:1575089	rs61423551_A	G	[+]	I	0.81
Brain iron § (neurodegeneration)	[13]	*FTH1*	11:61765133	rs10736716_G	C	[+]	I	0.96
Brain iron § (neurodegeneration)	[13]	*TMPRSS6*	22:37469192	rs6000553_G	A	[+]	I	0.95
BMI (neurodegeneration)	[14]	*SEC16B*	1:177889480	rs543874_G	A	0.05	T/I	1.00
BMI (neurodegeneration)	[14]	*NEGR1*	1:72751185	rs3101336_C	T	0.03	T/I	1.00
BMI (neurodegeneration)	[14]	*FPGT-TNNI3K*	1:75002193	rs12566985_G	A	0.02	T/I	1.00
BMI (neurodegeneration)	[14]	*PTBP2*	1:96924097	rs11165643_T	C	0.02	T/I	1.00
BMI (neurodegeneration)	[14]	*FUBP1*	1:78446761	rs12401738_A	G	0.02	T/I	1.00
BMI (neurodegeneration)	[14]	*NAV1*	1:201784287	rs2820292_C	A	0.02	T/I	1.00
BMI (neurodegeneration)	[14]	*AGBL4*	1:49589847	rs657452_A	G	0.02	T/I	1.00
BMI (neurodegeneration)	[14]	*ELAVL4*	1:50559820	rs11583200_C	T	0.02	T/I	1.00
BMI (neurodegeneration)	[14]	*TMEM18*	2:632348	rs13021737_G	A	0.06	T/I	1.00
BMI (neurodegeneration)	[14]	*ADCY3*	2:25150296	rs10182181_G	A	0.03	T/I	1.00
BMI (neurodegeneration)	[14]	*LINC01122*	2:59305625	rs1016287_T	C	0.02	T/I	1.00
BMI (neurodegeneration)	[14]	*EHBP1*	2:63053048	rs11688816_G	A	0.02	T/I	1.00
BMI (neurodegeneration)	[14]	*ERBB4*	2:213413231	rs7599312_G	A	0.02	T/I	1.00
BMI (neurodegeneration)	[14]	*UBE2E3*	2:181550962	rs1528435_T	C	0.02	T/I	1.00
BMI (neurodegeneration)	[14]	*LRP1B*	2:143043285	rs2121279_T	C	0.03	T/I	1.00
BMI (neurodegeneration)	[14]	*KCNK3*	2:26928811	rs11126666_A	G	0.02	T/I	1.00
BMI (neurodegeneration)	[14]	*ETV5*	3:185824004	rs1516725_C	T	0.05	T/I	1.00
BMI (neurodegeneration)	[14]	*RASA2*	3:141275436	rs16851483_T	G	0.05	T/I	1.00
BMI (neurodegeneration)	[14]	*CADM2*	3:85807590	rs13078960_G	T	0.03	T/I	1.00
BMI (neurodegeneration)	[14]	*FHIT*	3:61236462	rs2365389_C	T	0.02	T/I	1.00
BMI (neurodegeneration)	[14]	*RARB*	3:25106437	rs6804842_G	A	0.02	T/I	1.00
BMI (neurodegeneration)	[14]	*GBE1*	3:81792112	rs3849570_A	C	0.02	I	0.97
BMI (neurodegeneration)	[14]	*GNPDA2*	4:45182527	rs10938397_G	A	0.04	T/I	1.00

Trait (MIND component target)	Ref	Closest *Gene*	Chr:Pos GRCh37	SNP_EA	OA	Effect β	SNP Type*	R2†
BMI (neurodegeneration)	[14]	*SCARB2*	4:77129568	rs17001654_G	C	0.03	T/I	1.00
BMI (neurodegeneration)	[14]	*SLC39A8*	4:103188709	rs13107325_T	C	0.05	T/I	0.99
BMI (neurodegeneration)	[14]	*HHIP*	4:145659064	rs11727676_T	C	0.04	T/I	1.00
BMI (neurodegeneration)	[14]	*POC5*	5:75015242	rs2112347_T	G	0.03	T/I	1.00
BMI (neurodegeneration)	[14]	*TFAP2B*	6:50845490	rs2207139_G	A	0.05	T/I	1.00
BMI (neurodegeneration)	[14]	*PARK2*	6:163033350	rs13191362_A	G	0.03	T/I	1.00
BMI (neurodegeneration)	[14]	*C6orf106*	6:34563164	rs205262_G	A	0.02	T/I	1.00
BMI (neurodegeneration)	[14]	*TDRG1*	6:40348653	rs2033529_G	A	0.02	I	0.95
BMI (neurodegeneration)	[14]	*FOXO3*	6:108977663	rs9400239_C	T	0.02	T/I	1.00
BMI (neurodegeneration)	[14]	*PMS2L11*	7:76608143	rs2245368_C	T	0.03	I	0.86
BMI (neurodegeneration)	[14]	*HIP1*	7:75163169	rs1167827_G	A	0.02	T/I	1.00
BMI (neurodegeneration)	[14]	*HNF4G*	8:76806584	rs17405819_T	C	0.02	T/I	1.00
BMI (neurodegeneration)	[14]	*RALYL*	8:85079709	rs2033732_C	T	0.02	T/I	0.99
BMI (neurodegeneration)	[14]	*LINGO2*	9:28414339	rs10968576_G	A	0.03	T/I	1.00
BMI (neurodegeneration)	[14]	*LMX1B*	9:129460914	rs10733682_A	G	0.02	T/I	1.00
BMI (neurodegeneration)	[14]	*TLR4*	9:120378483	rs1928295_T	C	0.02	T/I	1.00
BMI (neurodegeneration)	[14]	*EPB41L4B*	9:111932342	rs6477694_C	T	0.02	T/I	1.00
BMI (neurodegeneration)	[14]	*C9orf93*	9:15634326	rs4740619_T	C	0.02	T/I	1.00
BMI (neurodegeneration)	[14]	*HIF1AN*	10:102395440	rs17094222_C	T	0.03	T/I	1.00
BMI (neurodegeneration)	[14]	*GRID1*	10:87410904	rs7899106_G	A	0.04	T/I	1.00
BMI (neurodegeneration)	[14]	*NT5C2*	10:104869038	rs11191560_C	T	0.03	T/I	1.00
BMI (neurodegeneration)	[14]	*TCF7L2*	10:114758349	rs7903146_C	T	0.02	T/I	1.00
BMI (neurodegeneration)	[14]	*BDNF*	11:27684517	rs11030104_A	G	0.04	T/I	1.00
BMI (neurodegeneration)	[14]	*MTCH2*	11:47650993	rs3817334_T	C	0.03	T/I	1.00
BMI (neurodegeneration)	[14]	*TRIM66*	11:8673939	rs4256980_G	C	0.02	T/I	1.00
BMI (neurodegeneration)	[14]	*CADM1*	11:115022404	rs12286929_G	A	0.02	T/I	1.00
BMI (neurodegeneration)	[14]	*HSD17B12*	11:43864278	rs2176598_T	C	0.02	T/I	1.00
BMI (neurodegeneration)	[14]	*BCDIN3D*	12:50247468	rs7138803_A	G	0.03	T/I	1.00
BMI (neurodegeneration)	[14]	*CLIP1*	12:122781897	rs11057405_G	A	0.03	T/I	1.00
BMI (neurodegeneration)	[14]	*MTIF3*	13:28017782	rs9581854_T	C	0.03	T/I	1.00
BMI (neurodegeneration)	[14]	*OLFM4*	13:54102206	rs12429545_A	G	0.03	T/I	1.00
BMI (neurodegeneration)	[14]	*NRXN3*	14:79899454	rs7141420_T	C	0.02	T/I	1.00
BMI (neurodegeneration)	[14]	*PRKD1*	14:29736838	rs12885454_C	A	0.02	T/I	1.00
BMI (neurodegeneration)	[14]	*STXBP6*	14:25928179	rs10132280_C	A	0.02	T/I	1.00
BMI (neurodegeneration)	[14]	*MAP2K5*	15:68077168	rs16951275_T	C	0.03	T/I	1.00
BMI (neurodegeneration)	[14]	*DMXL2*	15:51748610	rs3736485_A	G	0.02	T/I	1.00
BMI (neurodegeneration)	[14]	*FTO*	16:53803574	rs1558902_A	T	0.08	I	0.96
BMI (neurodegeneration)	[14]	*GPRC5B*	16:19935389	rs12446632_G	A	0.04	T/I	1.00
BMI (neurodegeneration)	[14]	*ATP2A1*	16:28889486	rs3888190_A	C	0.03	I	0.92
BMI (neurodegeneration)	[14]	*SBK1*	16:28333411	rs2650492_A	G	0.02	T/I	1.00
BMI (neurodegeneration)	[14]	*KAT8*	16:31129895	rs9925964_A	G	0.02	T/I	1.00
BMI (neurodegeneration)	[14]	*NLRC3*	16:3627358	rs758747_T	C	0.02	I	0.81
BMI (neurodegeneration)	[14]	*RPTOR*	17:78615571	rs12940622_G	A	0.02	I	0.96
BMI (neurodegeneration)	[14]	*RABEP1*	17:5283252	rs1000940_G	A	0.02	T/I	1.00
BMI (neurodegeneration)	[14]	*MC4R*	18:57829135	rs6567160_C	T	0.02	T/I	1.00
BMI (neurodegeneration)	[14]	*C18orf8*	18:21104888	rs1808579_C	T	0.02	T/I	1.00
BMI (neurodegeneration)	[14]	*GRP*	18:56883319	rs7243357_T	G	0.02	T/I	1.00
BMI (neurodegeneration)	[14]	*ZC3H4*	19:47569003	rs3810291_A	G	0.03	T/I	1.00
BMI (neurodegeneration)	[14]	*QPCTL*	19:46202172	rs2287019_C	T	0.04	T/I	1.00
BMI (neurodegeneration)	[14]	*KCTD15*	19:34309532	rs29941_G	A	0.02	T/I	1.00
BMI (neurodegeneration)	[14]	*PGPEP1*	19:18454825	rs17724992_A	G	0.02	T/I	1.00
BMI (neurodegeneration)	[14]	*TOMM40*	19:45395619	rs2075650_A	G	0.03	T/I	1.00
IR/FI (neurodegeneration)	[15]	*COBLL1*	2:165518799	rs75265117_C	G	0.03	I	0.96
IR/FI (neurodegeneration)	[15]	*COBLL1*	2:165528876	rs13389219_C	T	0.02	T/I	1.00
IR/FI (neurodegeneration)	[15]	*LOC646736*	2:227099534	rs2943646_G	A	0.03	I	0.95
IR/FI (neurodegeneration)	[15]	*SYN2*	3:12116620	rs308971_G	A	0.02	T/I	1.00
IR/FI (neurodegeneration)	[15]	*PPARG*	3:12351521	rs35000407_T	G	0.03	I	0.99
IR/FI (neurodegeneration)	[15]	*FAM13A*	4:89739808	rs3775380_G	A	0.01	T/I	1.00

Trait (MIND component target)	Ref	Closest *Gene*	Chr:Pos GRCh37	SNP_EA	OA	Effect β	SNP Type*	R2†
IR/FI (neurodegeneration)	[15]	*TET2*	4:106081636	rs9884482_C	T	0.01	T/I	1.00
IR/FI (neurodegeneration)	[15]	*HHIP*	4:145659064	rs11727676_C	T	0.02	T/I	1.00
IR/FI (neurodegeneration)	[15]	*LOC101928448*	5:55860781	rs3936511_G	A	0.02	I	0.97
IR/FI (neurodegeneration)	[15]	*C6orf1*	6:34222201	rs116141873_T	G	0.04	I	0.98
IR/FI (neurodegeneration)	[15]	*RPS10*	6:34236973	rs2780215_A	G	0.04	I	0.84
IR/FI (neurodegeneration)	[15]	*LINC01512*	6:43809802	rs9472135_T	C	0.01	I	0.96
IR/FI (neurodegeneration)	[15]	*RSPO3*	6:127449246	rs1474696_G	A	0.01	I	0.94
IR/FI (neurodegeneration)	[15]	*QKI*	6:164126233	rs73013411_C	A	0.02	I	0.89
IR/FI (neurodegeneration)	[15]	*GRB10*	7:50786663	rs2108349_G	A	0.01	T/I	1.00
IR/FI (neurodegeneration)	[15]	*KLF14*	7:130466854	rs972283_G	A	0.01	T/I	1.00
IR/FI (neurodegeneration)	[15]	*PPP1R3B*	8:9021933	rs330945_T	C	0.01	I	0.87
IR/FI (neurodegeneration)	[15]	*LOC157273*	8:9183596	rs4841132_A	G	0.03	T/I	1.00
IR/FI (neurodegeneration)	[15]	*NKX2-6*	8:23615445	rs13258890_T	C	0.01	I	0.95
IR/FI (neurodegeneration)	[15]	*ABO*	9:136132954	rs75179845_C	T	0.02	I	0.98
IR/FI (neurodegeneration)	[15]	*PTEN*	10:89680631	rs118164457_C	T	0.03	I	0.93
IR/FI (neurodegeneration)	[15]	*MACROD1*	11:63869062	rs2845885_C	T	0.02	T/I	1.00
IR/FI (neurodegeneration)	[15]	*GYS2*	12:21699928	rs6487237_A	C	0.02	I	0.94
IR/FI (neurodegeneration)	[15]	*HMGA2*	12:66351826	rs1351394_C	T	0.01	T/I	1.00
IR/FI (neurodegeneration)	[15]	*IGF1*	12:102740822	rs1402013_G	A	0.01	I	0.97
IR/FI (neurodegeneration)	[15]	*IGF1*	12:102898446	rs860598_A	G	0.02	I	**0.78**
IR/FI (neurodegeneration)	[15]	*DNAH10*	12:124409502	rs7133378_G	A	0.01	T/I	1.00
IR/FI (neurodegeneration)	[15]	*PEPD*	19:33899065	rs731839_G	A	0.01	T/I	1.00
IR/FI (neurodegeneration)	[15]	*EYA2*	20:45582472	rs1206760_G	A	0.01	I	0.91

EA: effect allele, OA: other allele; IR: insulin resistance, FI: fasting insulin;
[-]: GS were not weighted when effect sizes were not published, not comparable (i.e. different GWAS) or based on a single SNP; in these cases effects sizes were assumed =1 (increase)
*SNP Type: imputed (I), typed (T)
†Imputation quality metric (SNPs with R2<0.8 were not included in GS)
‡Strong and plausible but not P<5E-8
§From Table 3 (brain MS/MS, P<5E-8) and Table 4 (brain QSM/T2*, P<5E-8) in Ref [13]

Table S3. Cognitive domains and tests used in the MIND Trial

**Domain: Episodic Memory**
Word List Memory
Word List Recall
Word List Recognition
East Boston Story Immediate
East Boston Story Delayed
**Domain: Semantic Memory**
Multilingual Naming Test
Verbal / Category Fluency
**Domain: Perceptual Speed**
Oral Digit Symbol test (NIH)
Pattern Comparison (NIH)
Trail Making Test A
**Domain: Executive Function**
Trail Making Test B
Flanker Inhibitory control (NIH)

The three main executive functions are working memory, cognitive flexibility and inhibition control

Table S4. Late-Onset Alzheimer's Disease Risk Loci

Closest Gene	Chr:Pos GRCh37	SNP_EA	OA	EAF		Effect OR*	Effect β	GS_{AD}	GS_{AD-I}	GS_{AD-C}
				EUR	AFR					
CR1	1:207692049	rs6656401_A	G	0.20	0.01	1.18	0.16	x	x	
BIN1	2:127892810	rs6733839_T	C	0.41	0.42	1.22	0.19	x		
INPP5D	2:234068476	rs35349669_T	C	0.48	0.10	1.08	0.07	x	x	
CLNK	4:11026028	rs6448453_A	G	0.25	0.10	1.09	0.09	x	x	
MEF2C	5:88223420	rs190982_A	G	0.63	0.92	1.08	0.08	x	x	
HLA-DRB1/5	6:32578530	rs9271192_C (rs111418223_C)	A	0.27	0.23	1.11	0.10	x	x	
CD2AP	6:47487762	rs10948363_G	A	0.27	0.27	1.1	0.10	x		
NME8	7:37841534	rs2718058_A	G	0.64	0.49	1.08	0.07	x		
ZCWPW1	7:100004446	rs1476679_T	C	0.69	0.98	1.1	0.08	x		
EPHA1	7:143110762	rs11771145_G	A	0.63	0.43	1.11	0.10	x	x	
PTK2B	8:27195121	rs28834970_C	T	0.35	0.22	1.1	0.10	x		
CLU	8:27467686	rs9331896_T	C	0.60	0.38	1.16	0.15	x	x	x
ECHDC3	10:11720308	rs7920721_G	A	0.36	0.11	1.08	0.08	x		
CELF1	11:47557871	rs10838725_C	T	0.27	0.02	1.08	0.08	x		
MS4A6A	11:59923508	rs983392_A	G	0.58	0.97	1.16	0.11	x	x	
PICALM	11:85867875	rs10792832_G	A	0.62	0.91	1.34	0.13	x		
SORL1	11:121435587	rs11218343_T	C	0.96	0.10	1.3	0.27	x		x
FERMT2	14:53400629	rs17125944_C	T	0.08	0.06	1.14	0.12	x		
SLC24A4	14:92926952	rs10498633_G	T	0.79	0.88	1.01	0.10	x		x
ADAM10	15:59045774	rs593742_A	G	0.64	0.21	1.04	0.08	x		
IQCK	16:19808163	rs7185636_T	C	0.85	0.09	1.09	0.09	x		
ABCA7	19:1063443	rs4147929_A	G	0.17	0.02	1.22	0.13	x		x
CD33	19:51727962	rs3865444_C	A	0.69	0.93	1.10	0.10	x	x	
CASS4	20:55018260	rs7274581_T	C	0.92	0.73	1.14	0.14	x		
ADAMTS1	21:28156856	rs2830500_C	A	0.70	0.91	1.07	0.07	x	x	

AFR: African ancestry, EA: effect allele, EAF: EA frequency, EUR: European ancestry, GS_{AD}: AD predisposition genetic score (I: immune response; C: cholesterol metabolism), OA: other allele; OR: odds ratio. *As reported by the two largest GWAS to date[16,17].

Table S5. Nominal associations (0.004<P<0.05) between genetic scores (GS) and baseline cognitive performance

Genetic scores	N	Estimated mean global function (95% CI)	Estimated mean executive function (95% CI)	Estimated mean semantic function (95% CI)	Beta (standard error)	P value
GS_{BMI}						
Tertile1	164	-0.008 (-0.08,0.07)			0.009 (0.004)	0.03
Tertile2	165	0.03 (-0.04-0.11)	n.s	n.s		
Tertile3	165	0.12 (0.05,0.20)				
$GS_{azelaic}$ (rs1165213)						
GG	106		-0.04 (-0.18,0.10)		0.10 (0.05)	0.04
AG	242	n.s	0.10 (0.01,0.19)	n.s		
AA	146		0.17 (0.05,0.28)			
GS_{COMT} (rs4680)						
AA[Met/Met]	134		0.20 (0.08,0.32)		-0.10 (0.05)	0.03
AG[Met/Val]	242	n.s	0.08 (-0.01,0.17)	n.s		
GG[Val/Val]	118		-0.004 (-0.13,0.13)			
GS_{folate} (rs1801133)						
AA[Val/Val]	75			-0.12 (-0.29,0.04)	0.11 (0.05)	0.02
AG[Val/Ala]	213	n.s	n.s	0.13 (0.03,0.22)		
GG[Ala/Ala]	206			0.14 (0.04,0.24)		

n.s. not significant (P>0.05)

Table S6. Estimated mean change (95% CI) in executive function from baseline by $GS_{azelaic}$ and GS_{COMT}

Years since randomization	$GS_{azelaic}$ (rs1165213)			GS_{COMT} (rs4680)		
	GG	AG	AA	AA[Met/Met]	AG[Met/Val]	GG[Val/Val]
Baseline	Ref	Ref	Ref	Ref	Ref	Ref
0.5	0.08 (-0.02, 0.18)	0.10 (0.03, 0.17)	0.07 (-0.004, 0.14)	0.09 (-0.001, 0.18)	0.05 (-0.02, 0.11)	0.16 (0.07, 0.26)
1.0	0.21 (0.09, 0.33)	0.13 (0.06, 0.20)	0.07 (-0.02, 0.16)	0.13 (0.03, 0.23)	0.11 (0.04, 0.19)	0.15 (0.06, 0.25)
2.0	0.27 (0.10, 0.43)	0.12 (0.05, 0.19)	0.04 (-0.06, 0.14)	0.07 (-0.05, 0.18)	0.14 (0.06, 0.23)	0.17 (0.06, 0.25)
3.0	0.24 (0.08, 0.40)	0.11 (0.03, 0.18)	0.05 (-0.04, 0.14)	-0.01 (-0.12, 0.09)	0.15 (0.07, 0.23)	0.19 (0.09, 0.30)
N per time	97/93/92/86	227/223/224/213	142/141/136/131	129/128/129/115	229/221/218/213	108/108/105/102

Table S7. Estimated mean change (95% CI) in perceptual speed from baseline by diet assignment and GS_{COMT}

Years since randomization	Control			MIND		
	AA[Met/Met]	AG[Met/Val]	GG[Val/Val]	AA[Met/Met]	AG[Met/Val]	GG[Val/Val]
Baseline	Ref	Ref	Ref	Ref	Ref	Ref
0.5	-0.07 (-0.20, 0.05)	-0.03 (-0.13, 0.07)	0.19 (0.07, 0.31)	0.20 (0.08, 0.33)	0.13 (0.04, 0.22)	-0.04 (-0.16, 0.09)
1.0	0.12 (-0.01, 0.25)	0.17 (0.08, 0.26)	0.25 (0.10, 0.41)	0.28 (0.17, 0.39)	0.29 (0.21, 0.37)	0.08 (-0.03, 0.19)
2.0	0.02 (-0.11, 0.14)	0.22 (0.14, 0.31)	0.33 (0.23, 0.44)	0.29 (0.17, 0.41)	0.26 (0.16, 0.35)	0.13 (-0.003, 0.27)
3.0	0.03 (-0.13, 0.18)	0.23 (0.07, 0.40)	0.23 (0.07, 0.40)	0.18 (0.07, 0.30)	0.15 (0.04, 0.25)	0.11 (-0.04, 0.25)
N per time point	64/63/64/57	113/112/112/111	55/53/53/53	66/66/65/59	116/109/106/102	53/55/52/49

Table S8. Genome-wide SNP×diet interactions and change in cognitive function after 3 years

Cognitive Function Domain	Chr:Pos	SNP dbSNP	EA	OA	EAF	Method 1 (Slope)			Method 2 (Change from baseline)			Imputed	R2	Type	Closest Gene
						β interaction	SE	P-value	β interaction	SE	P-value				
executive	1:82601906	rs551741322	C	A	0.04	0.39	0.07	4.5E-08	0.55	0.11	1.5E-06	Yes	0.95	intergenic	ADGRL2
executive	1:193681501	rs144408988	C	T	0.02	0.65	0.11	6.0E-09	0.85	0.18	2.8E-06	Yes	0.90	intron	LOC124904475
executive	1:195952622	rs190731023	G	A	0.01	0.91	0.14	3.3E-10	1.12	0.23	1.7E-06	Yes	0.82	intergenic	KCNT2
executive	1:220083020	rs116894725	T	C	0.01	1.77	0.20	1.3E-17	2.07	0.33	1.4E-09	Yes	0.85	intergenic	SLC30A10
executive	1:226332087	rs114166853	A	G	0.01	1.27	0.19	3.1E-11	1.48	0.31	2.4E-06	Yes	0.81	3' UTR	ACBD3
executive	3:148774828	rs574786788	T	C	0.01	0.70	0.13	3.9E-08	1.12	0.21	1.0E-07	Yes	0.99	intron	HLTF
executive	4:169570129	rs12331897	G	A	0.02	0.59	0.10	1.4E-08	0.86	0.17	3.0E-07	No	0.98	intron	PALLD
executive	5:8747056	rs74809373	G	C	0.01	0.93	0.13	2.3E-12	1.38	0.21	9.0E-11	Yes	0.89	intergenic	SEMA5A
executive	6:130789713	rs9492603	T	C	0.02	0.98	0.15	1.8E-10	1.33	0.25	1.0E-07	Yes	0.84	intergenic	TMEM200A
executive	8:75001294	rs114134891	G	A	0.01	1.00	0.15	3.9E-11	1.20	0.24	7.8E-07	Yes	0.82	intron	LY96
executive	8:80112029	rs13256135	C	T	0.01	0.93	0.15	3.1E-09	1.23	0.25	1.6E-06	Yes	0.85	intergenic	STMN2
executive	11:78940749	rs143703635	T	G	0.01	1.02	0.15	9.2E-11	1.26	0.25	9.0E-07	Yes	0.84	intron	TENM4
executive	11:88516919	rs11825286	T	C	0.01	1.81	0.20	3.6E-18	2.02	0.33	3.0E-09	Yes	0.97	intron	GRM5
executive	12:11825291	rs2724649	A	G	0.02	0.52	0.10	1.8E-07	0.95	0.16	6.1E-09	No	1.00	intron	ETV6
executive	12:117954413	rs11068525	G	C	0.01	0.78	0.13	1.0E-09	1.02	0.20	6.8E-07	Yes	0.85	intron	KSR2
executive	13:109534647	rs76159459	G	A	0.01	0.86	0.15	3.6E-08	1.27	0.25	4.8E-07	Yes	0.85	intron	MYO16
executive	14:40146923	rs76700247	G	T	0.01	0.81	0.11	5.7E-12	1.08	0.19	1.4E-08	Yes	0.88	intron	LOC105370461
executive	18:58787680	rs17067805	T	G	0.01	0.95	0.15	5.0E-10	1.23	0.24	5.8E-07	No	1.00	intergenic	CDH20
global	20:42194802	rs6065627	G	A	0.10	0.13	0.02	4.7E-09	0.19	0.04	7.0E-07	No	1.00	intron	SGK2
perceptual	18:73136235	rs71359090	G	C	0.01	-0.81	0.12	1.2E-10	-0.94	0.20	4.6E-06	Yes	0.96	intron	SMIM21
semantic	1:61771758	rs187224681	A	G	0.01	-0.59	0.12	1.1E-06	-1.30	0.23	1.6E-08	Yes	0.96	intron	NFIA
semantic	2:23446116	rs184902652	A	C	0.01	-0.61	0.11	3.4E-08	-1.18	0.20	1.2E-08	Yes	0.95	intergenic	KLHL29
semantic	3:151333979	rs142160392	T	C	0.01	-1.04	0.16	3.0E-10	-2.18	0.30	2.4E-12	Yes	0.97	intron	IGSF10
semantic	4:185735318	rs183299944	G	T	0.01	-0.78	0.13	1.2E-08	-1.51	0.25	2.6E-09	Yes	0.90	intron	ACSL1
semantic	9:15537505	rs117193613	G	A	0.01	-1.09	0.17	1.3E-10	-2.21	0.31	3.9E-12	No	0.98	intergenic	CCDC171
semantic	10:33233000	rs147566745	A	G	0.01	-0.59	0.13	3.7E-06	-1.33	0.23	1.9E-08	Yes	0.88	intron	ITGB1
semantic	10:114124977	rs74563318	A	C	0.02	-0.38	0.07	6.1E-08	-0.81	0.13	9.8E-10	Yes	0.91	intergenic	ACSL5
semantic	10:120204509	rs182818217	T	C	0.01	-0.70	0.13	4.4E-08	-1.40	0.24	6.4E-09	Yes	0.82	intergenic	PRLHR
semantic	16:62758806	rs190940994	T	C	0.01	-0.46	0.09	5.9E-07	-0.96	0.17	3.4E-08	Yes	0.90	5'UTR	LOC102723560

Table S9. Estimated mean change (SD) in cognitive scores at 3 years from baseline stratified by genotype and diet assignment

Cognitive Function Domain	SNP_EA= # EAs	Control			MIND		
		Mean	SD	N	Mean	SD	N
Semantic	rs190940994_C=0	4.63	.	1	.	.	0
	rs190940994_C=1	0.45	0.77	3	-0.07	0.61	6
	rs190940994_C=2	0.14	0.59	241	0.14	0.54	243
	rs187224681_G=0	.	.	0	-0.50	.	1
	rs187224681_G=1	2.28	3.32	2	-0.15	0.58	6
	rs187224681_G=2	0.14	0.60	243	0.14	0.54	242
	rs184902652_C=0	.	.	0	.	.	0
	rs184902652_C=1	2.28	2.09	3	0.00	0.60	7
	rs184902652_C=2	0.13	0.59	242	0.13	0.54	242
	rs183299944_T=0	.	.	0	.	.	0
	rs183299944_T=1	2.88	2.47	2	0.22	0.55	8
	rs183299944_T=2	0.14	0.59	243	0.13	0.54	241
	rs182818217_C=0	.	.	0	.	.	0
	rs182818217_C=1	2.66	2.78	2	-0.06	0.55	7
	rs182818217_C=2	0.14	0.60	243	0.14	0.54	242
	rs147566745_G=0	.	.	0	.	.	0
	rs147566745_G=1	2.92	2.42	2	0.18	0.25	8
	rs147566745_G=2	0.14	0.59	243	0.13	0.55	241
	rs142160392_C=0	.	.	0	.	.	0
	rs142160392_C=1	4.63	.	1	0.27	0.37	11
	rs142160392_C=2	0.14	0.60	244	0.12	0.55	238
	rs117193613_A=0	.	.	0	.	.	0
	rs117193613_A=1	4.63	.	1	0.36	0.66	10
	rs117193613_A=2	0.14	0.60	244	0.12	0.54	239
	rs74563318_C=0	4.63	.	1	-1.36	.	1
	rs74563318_C=1	0.67	0.65	6	0.08	0.62	9
	rs74563318_C=2	0.13	0.59	238	0.14	0.53	239
Global	rs6065627_A=0	0.00	0.39	4	0.48	0.27	2
	rs6065627_A=1	0.03	0.43	39	0.37	0.38	46
	rs6065627_A=2	0.21	0.34	202	0.15	0.36	201
Perceptual Speed	rs71359090_C=0	.	.	0	.	.	0
	rs71359090_C=1	0.64	0.85	8	-1.21	1.37	2
	rs71359090_C=2	0.11	0.53	237	0.13	0.49	247
Executive	rs76700247_T=0	.	.	0	.	.	0
	rs76700247_T=1	-1.46	1.71	6	0.41	0.47	7
	rs76700247_T=2	0.10	0.55	239	0.09	0.62	242
	rs17067805_G=0	.	.	0	.	.	0
	rs17067805_G=1	-1.88	2.66	2	0.50	0.60	10
	rs17067805_G=2	0.07	0.59	243	0.09	0.62	239
	rs574786788_C=0	.	.	0	.	.	0
	rs574786788_C=1	-1.62	2.11	4	0.53	0.46	6
	rs574786788_C=2	0.09	0.56	241	0.09	0.62	243

	rs551741322_A=0	.	.	0	.	.	0
	rs551741322_A=1	-0.67	1.18	13	0.42	0.82	24
	rs551741322_A=2	0.10	0.57	232	0.07	0.59	225
	rs190731023_A=0	.	.	0	.	.	0
	rs190731023_A=1	-1.14	1.97	4	0.44	0.51	6
	rs190731023_A=2	0.08	0.59	241	0.09	0.62	243
	rs144408988_T=0	.	.	0	.	.	0
	rs144408988_T=1	-1.58	1.55	4	0.23	0.49	14
	rs144408988_T=2	0.09	0.58	241	0.09	0.63	235
	rs143703635_G=0	.	.	0	.	.	0
	rs143703635_G=1	-2.11	2.34	2	0.24	0.60	9
	rs143703635_G=2	0.08	0.59	243	0.10	0.62	240
	rs116894725_C=0	.	.	0	.	.	0
	rs116894725_C=1	-3.76	.	1	0.20	0.54	9
	rs116894725_C=2	0.07	0.59	244	0.10	0.62	240
	rs114166853_G=0	.	.	0	.	.	0
	rs114166853_G=1	-3.76	.	1	0.09	0.40	7
	rs114166853_G=2	0.07	0.59	244	0.10	0.62	242
	rs114134891_A=0	.	.	0	.	.	0
	rs114134891_A=1	-2.50	1.78	2	0.15	0.29	9
	rs114134891_A=2	0.08	0.59	243	0.10	0.63	240
	rs76159459_A=0	.	.	0	.	.	0
	rs76159459_A=1	-1.85	2.71	2	0.60	0.72	8
	rs76159459_A=2	0.07	0.59	243	0.09	0.61	241
	rs74809373_C=0	.	.	0	0.11	.	1
	rs74809373_C=1	-2.29	1.91	3	0.47	0.81	8
	rs74809373_C=2	0.09	0.56	242	0.09	0.61	240
	rs13256135_T=0	.	.	0	.	.	0
	rs13256135_T=1	-2.22	2.18	2	0.26	0.29	8
	rs13256135_T=2	0.08	0.59	243	0.10	0.63	241
	rs12331897_A=0	.	.	0	.	.	0
	rs12331897_A=1	-1.19	1.50	6	0.41	0.76	9
	rs12331897_A=2	0.09	0.58	239	0.09	0.61	240
	rs11825286_C=0	.	.	0	.	.	0
	rs11825286_C=1	-3.76	.	1	0.13	0.33	10
	rs11825286_C=2	0.07	0.59	244	0.10	0.63	239
	rs11068525_C=0	.	.	0	0.17	.	1
	rs11068525_C=1	-2.27	1.94	3	-0.09	0.38	4
	rs11068525_C=2	0.09	0.56	242	0.11	0.62	244
	rs9492603_C=0	.	.	0	.	.	0
	rs9492603_C=1	-2.11	2.33	2	0.41	0.74	13
	rs9492603_C=2	0.08	0.59	243	0.09	0.61	236
	rs2724649_A=0	0.09	0.56	238	0.08	0.61	240
	rs2724649_A=1	-1.16	1.64	7	0.71	0.70	9
	rs2724649_A=2	.	.	0	.	.	0

EA: effect allele

Figure S1. GS$_{azelaic}$ (*SLC17A1*, rs1165213) and change in executive function

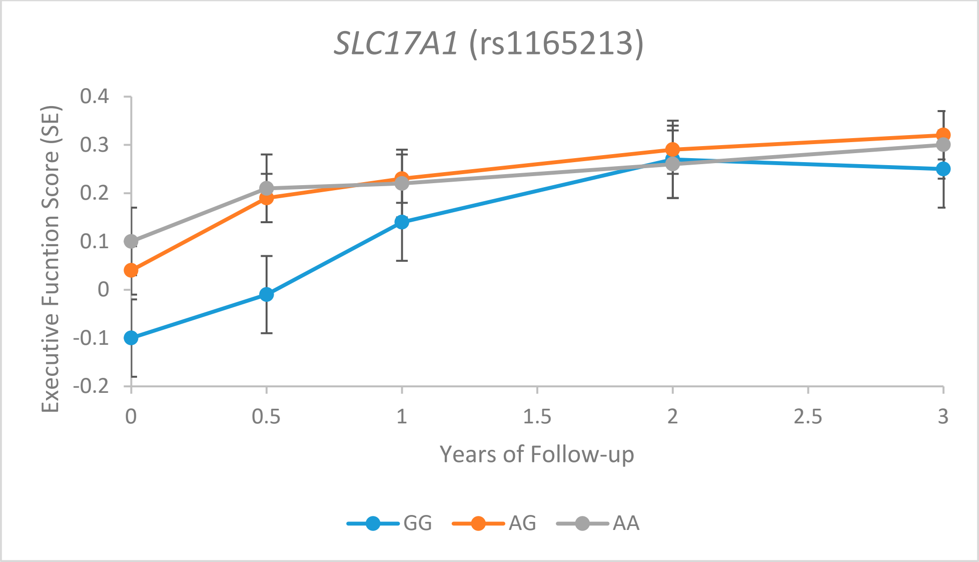

Shown are estimated means of executive function score by genotype from mixed linear models (P=0.009)

Figure S2. GS$_{COMT}$ (rs4680) and change in urinary tyrosol by diet assignment

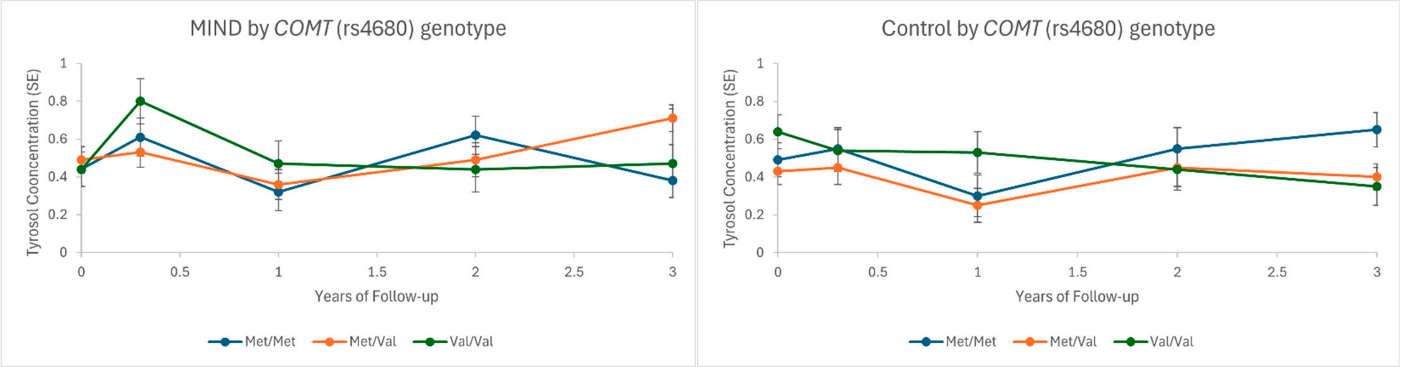

Shown are estimated means of urinary creatinine-adjusted tyrosol [tyrosol (ng/mL) / creatinine (mg/dL)] by genotype and diet assignment from linear mixed models (P=0.03 for interaction).

Figure S3. Manhattan plots for genome-wide association analysis of change in global cognition score

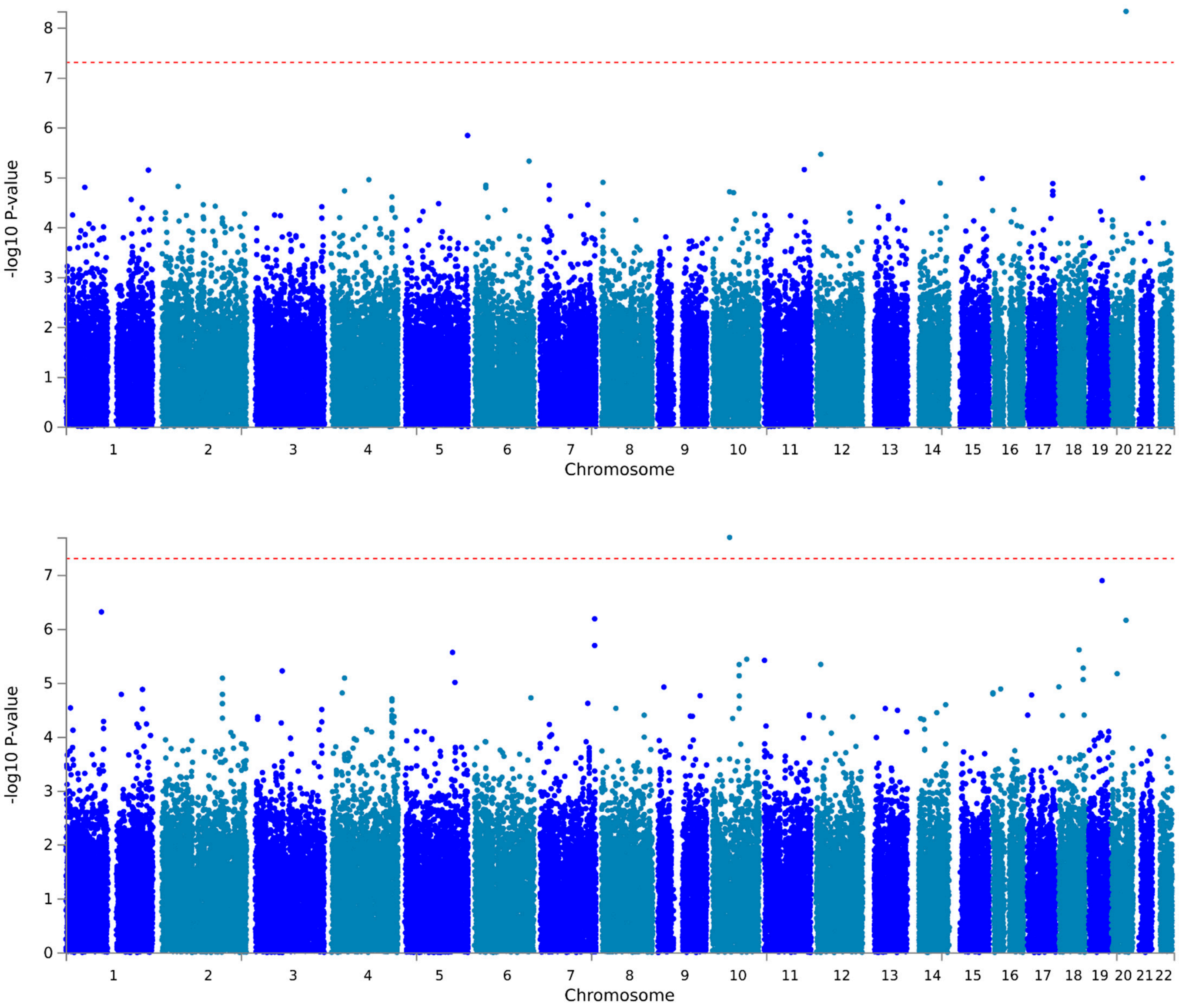

Results from mixed model approach (Top) and change in score from baseline to 36 months (Bottom). See Methods for description of the two approaches.

Figure S4. Manhattan plots for genome-wide association analysis of change in executive function score

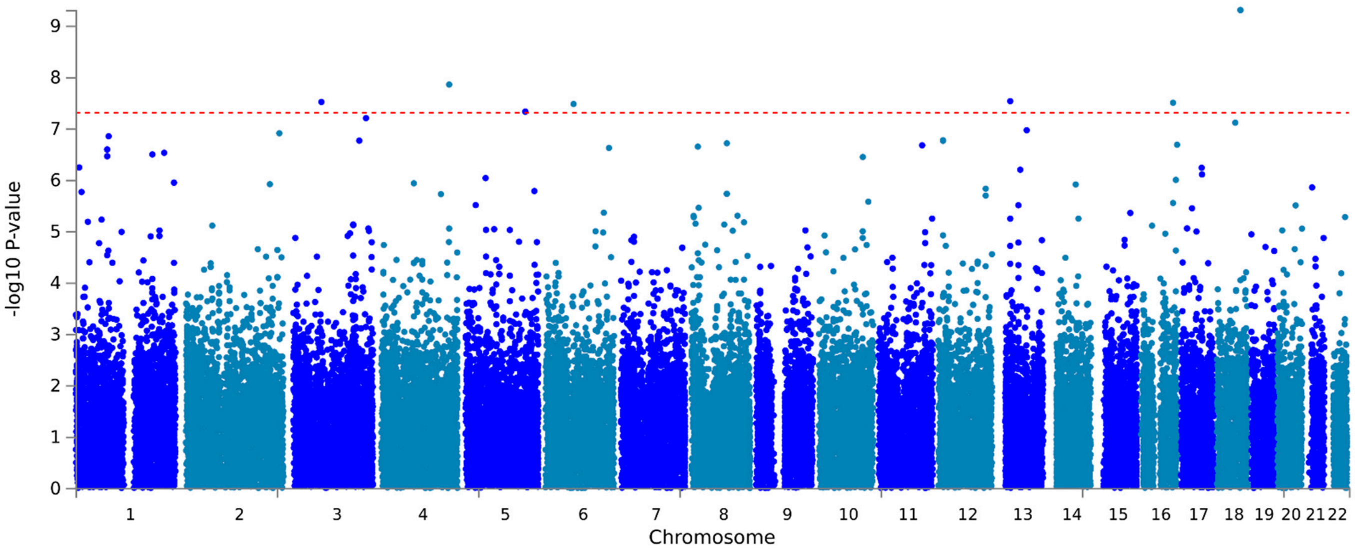

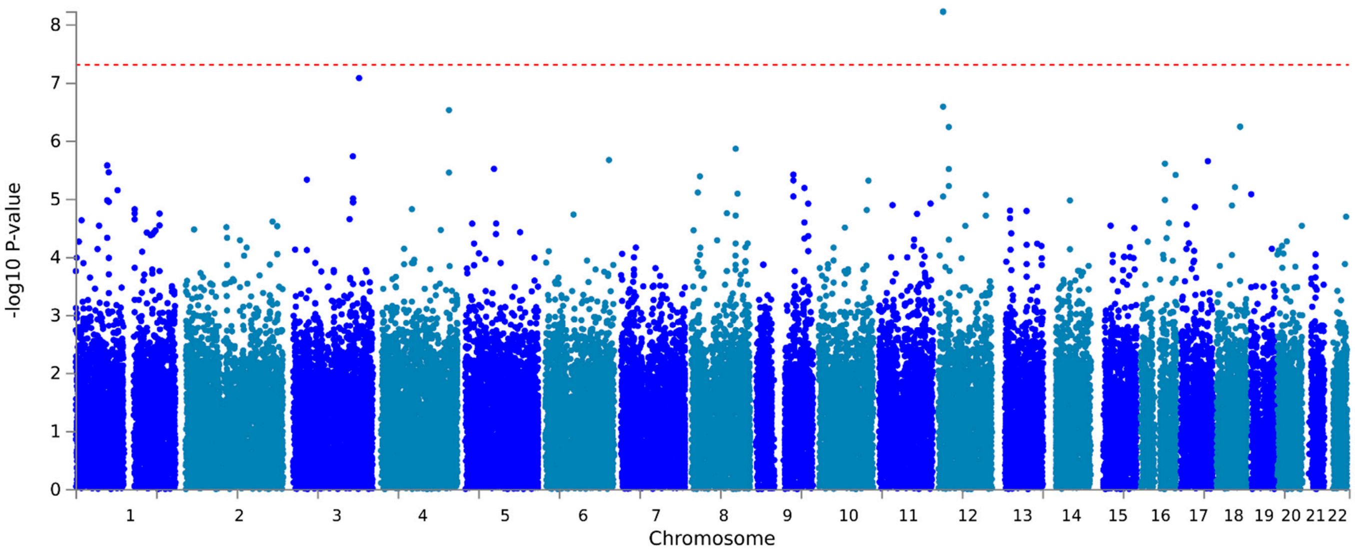

Results from mixed model approach (Top) and change in score from baseline to 36 months (Bottom). See Methods for description of the two approaches.

Figure S5. Manhattan plots for genome-wide association analysis of change in semantic memory score

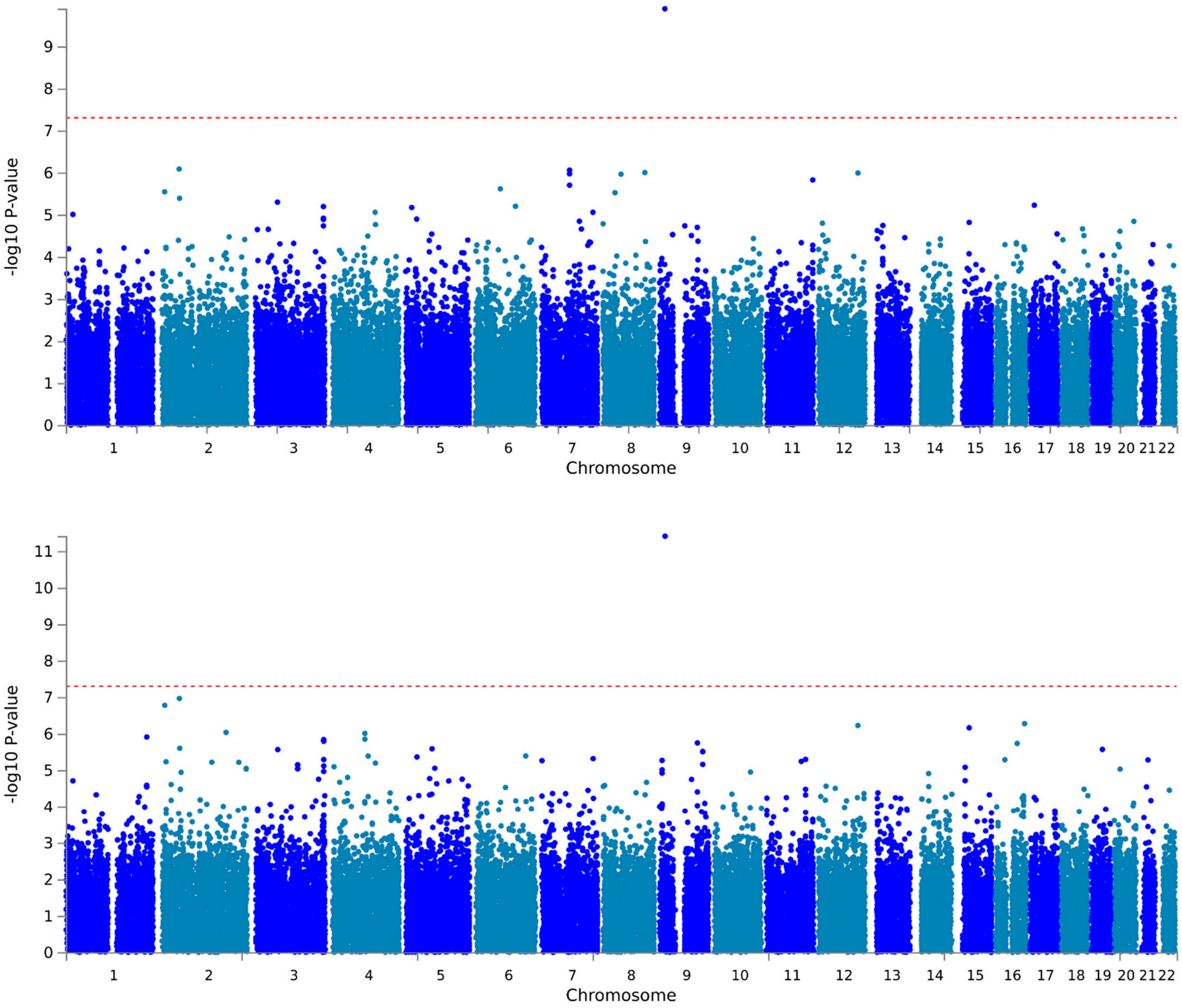

Results from mixed model approach (Top) and change in score from baseline to 36 months (Bottom). See Methods for description of the two approaches.

Figure S6. Manhattan plots for genome-wide association analysis of change in perceptual speed score

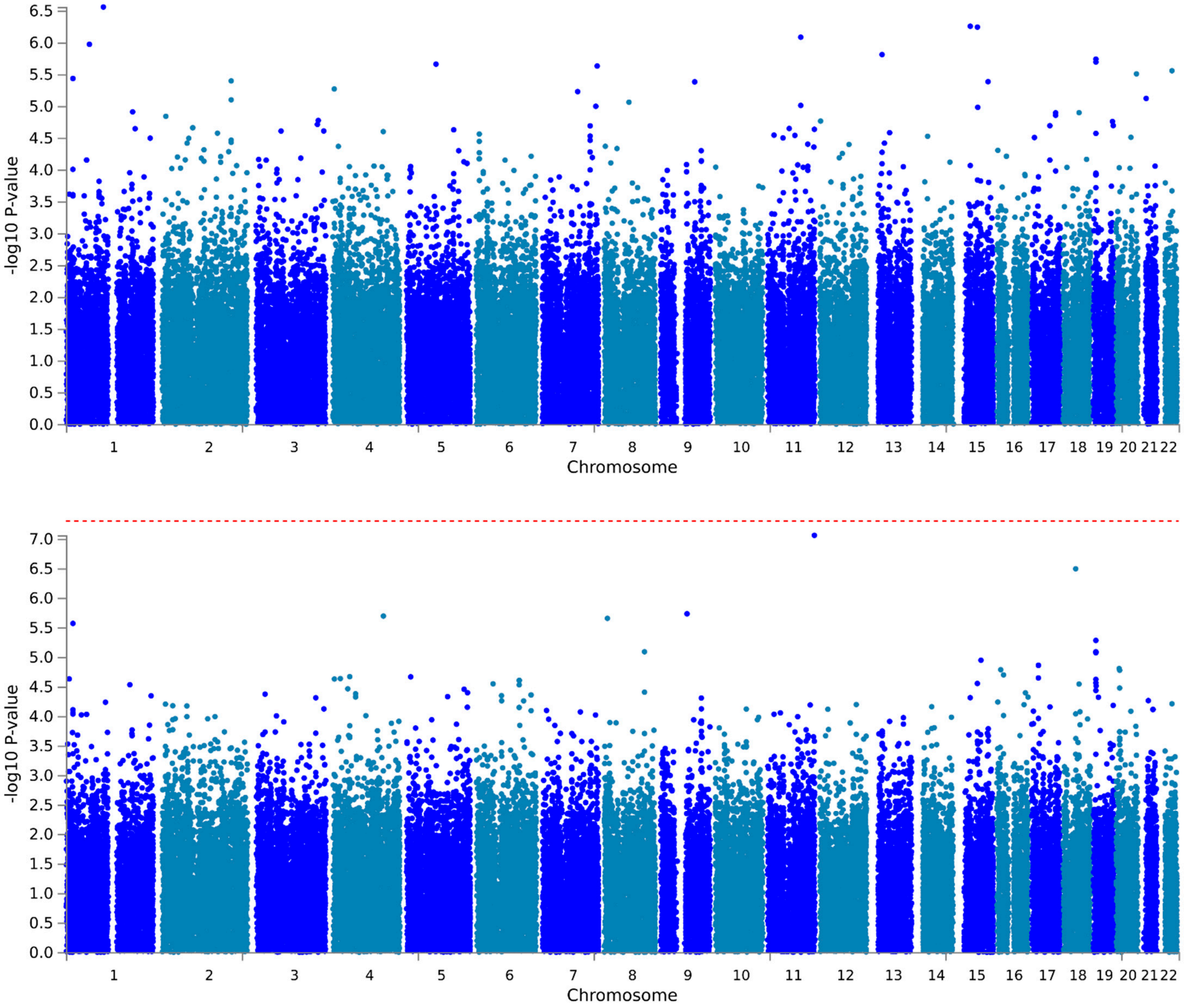

Results from mixed model approach (Top) and change in score from baseline to 36 months (Bottom). See Methods for description of the two approaches.

References

1. D'Adamo, C.R.; Dawson, V.J.; Ryan, K.A.; Yerges-Armstrong, L.M.; Semba, R.D.; Steinle, N.I.; Mitchell, B.D.; Shuldiner, A.R.; McArdle, P.F. The CAPN2/CAPN8 Locus on Chromosome 1q Is Associated with Variation in Serum Alpha-Carotene Concentrations. *J Nutrigenet Nutrigenomics* **2016**, *9*, 254-264, doi:10.1159/000452890.
2. Wood, A.R.; Perry, J.R.; Tanaka, T.; Hernandez, D.G.; Zheng, H.F.; Melzer, D.; Gibbs, J.R.; Nalls, M.A.; Weedon, M.N.; Spector, T.D., et al. Imputation of variants from the 1000 Genomes Project modestly improves known associations and can identify low-frequency variant-phenotype associations undetected by HapMap based imputation. *PLoS One* **2013**, *8*, e64343, doi:10.1371/journal.pone.0064343.
3. Major, J.M.; Yu, K.; Wheeler, W.; Zhang, H.; Cornelis, M.C.; Wright, M.E.; Yeager, M.; Snyder, K.; Weinstein, S.J.; Mondul, A., et al. Genome-wide association study identifies common variants associated with circulating vitamin E levels. *Hum Mol Genet* **2011**, *20*, 3876-3883.
4. Surendran, P.; Stewart, I.D.; Au Yeung, V.P.W.; Pietzner, M.; Raffler, J.; Worheide, M.A.; Li, C.; Smith, R.F.; Wittemans, L.B.L.; Bomba, L., et al. Rare and common genetic determinants of metabolic individuality and their effects on human health. *Nat Med* **2022**, *28*, 2321-2332, doi:10.1038/s41591-022-02046-0.
5. Grarup, N.; Sulem, P.; Sandholt, C.H.; Thorleifsson, G.; Ahluwalia, T.S.; Steinthorsdottir, V.; Bjarnason, H.; Gudbjartsson, D.F.; Magnusson, O.T.; Sparso, T., et al. Genetic architecture of vitamin B12 and folate levels uncovered applying deeply sequenced large datasets. *PLoS Genet* **2013**, *9*, e1003530, doi:10.1371/journal.pgen.1003530.
6. Dashti, H.S.; Shea, M.K.; Smith, C.E.; Tanaka, T.; Hruby, A.; Richardson, K.; Wang, T.J.; Nalls, M.A.; Guo, X.; Liu, Y., et al. Meta-analysis of genome-wide association studies for circulating phylloquinone concentrations. *Am J Clin Nutr* **2014**, *100*, 1462-1469, doi:10.3945/ajcn.114.093146.
7. Tunbridge, E.M. The catechol-O-methyltransferase gene: its regulation and polymorphisms. *Int Rev Neurobiol* **2010**, *95*, 7-27, doi:10.1016/b978-0-12-381326-8.00002-8.
8. Gonzalez-Dominguez, R.; Jauregui, O.; Queipo-Ortuno, M.I.; Andres-Lacueva, C. Characterization of the Human Exposome by a Comprehensive and Quantitative Large-Scale Multianalyte Metabolomics Platform. *Anal Chem* **2020**, *92*, 13767-13775, doi:10.1021/acs.analchem.0c02008.
9. Schlosser, P.; Scherer, N.; Grundner-Culemann, F.; Monteiro-Martins, S.; Haug, S.; Steinbrenner, I.; Uluvar, B.; Wuttke, M.; Cheng, Y.; Ekici, A.B., et al. Genetic studies of paired metabolomes reveal enzymatic and transport processes at the interface of plasma and urine. *Nat Genet* **2023**, *55*, 995-1008, doi:10.1038/s41588-023-01409-8.
10. Renai, L.; Ancillotti, C.; Ulaszewska, M.; Garcia-Aloy, M.; Mattivi, F.; Bartoletti, R.; Del Bubba, M. Comparison of chemometric strategies for potential exposure marker discovery and false-positive reduction in untargeted metabolomics: application to the serum analysis by LC-HRMS after intake of Vaccinium fruit supplements. *Anal Bioanal Chem* **2022**, *414*, 1841-1855, doi:10.1007/s00216-021-03815-5.
11. Bondia-Pons, I.; Barri, T.; Hanhineva, K.; Juntunen, K.; Dragsted, L.O.; Mykkänen, H.; Poutanen, K. UPLC-QTOF/MS metabolic profiling unveils urinary changes in humans after a whole grain rye versus refined wheat bread intervention. *Mol Nutr Food Res* **2013**, *57*, 412-422, doi:https://doi.org/10.1002/mnfr.201200571.
12. Hu, Y.; Tanaka, T.; Zhu, J.; Guan, W.; Wu, J.H.Y.; Psaty, B.M.; McKnight, B.; King, I.B.; Sun, Q.; Richard, M., et al. Discovery and fine-mapping of loci associated with MUFAs through trans-ethnic meta-analysis in Chinese and European populations. *J Lipid Res* **2017**, *58*, 974-981, doi:10.1194/jlr.P071860.
13. Cornelis, M.C.; Fazlollahi, A.; Bennett, D.A.; Schneider, J.A.; Ayton, S. Genetic Markers of Postmortem Brain Iron. *J Neurochem* **2025**, *169*, e16309, doi:10.1111/jnc.16309.

14. Locke, A.E.; Kahali, B.; Berndt, S.I.; Justice, A.E.; Pers, T.H.; Day, F.R.; Powell, C.; Vedantam, S.; Buchkovich, M.L.; Yang, J., et al. Genetic studies of body mass index yield new insights for obesity biology. *Nature* **2015**, *518*, 197-206, doi:10.1038/nature14177.
15. Chen, J.; Spracklen, C.N.; Marenne, G.; Varshney, A.; Corbin, L.J.; Luan, J.a.; Willems, S.M.; Wu, Y.; Zhang, X.; Horikoshi, M., et al. The trans-ancestral genomic architecture of glycemic traits. *Nat Genet* **2021**, *53*, 840-860, doi:10.1038/s41588-021-00852-9.
16. Lambert, J.C.; Ibrahim-Verbaas, C.A.; Harold, D.; Naj, A.C.; Sims, R.; Bellenguez, C.; Jun, G.; Destefano, A.L.; Bis, J.C.; Beecham, G.W., et al. Meta-analysis of 74,046 individuals identifies 11 new susceptibility loci for Alzheimer's disease. *Nat Genet* **2013**, *45*, 1452-1458.
17. Jansen, I.; Savage, J.; Watanabe, K.; Bryois, J.; Williams, D.; Steinberg, S.; Sealock, J.; Karlsson, I.; Hägg, S.; Athanasiu, L. Genome-wide meta-analysis identifies new loci and functional pathways influencing Alzheimer's disease risk. *Nat Genet* **2019**.
